# Supplementary material for: DW2009 Elevates the Efficacy of Donepezil against Cognitive Impairment in Mice
Source: Nutrients. 2021 Sep 19;13(9):3273. doi: 10.3390/nu13093273 (PMC8465226; doi:10.3390/nu13093273)
Supplement: Supplementary file 1 [file nutrients-13-03273-s001.zip › nutrients-1357173-supplementary.pdf]

[Supplementary material]

DW2009 elevates the efficacy of donepezil against cognitive decline in mice

Dong-Yun Lee, Jeon-Kyung Kim, Soo-Won Yun, Myung Joo Han, and Dong-Hyun Kim

Table S1. F and P values of all experimental data.

Figure1			
NC vs LPS, LPS vs C29, LPS vs DW			
(B)	Spon. Alternation(%)		F(1,10)=25.588, p<0.05$^{\#}$<br>F(1,10)=39.410, p<0.05*<br>F(1,10)=18.466, p<0.05*
(C)	Number of enteries		F(1,10)=0.049, p>0.05<br>F(1,10)=0.095, p>0.05<br>F(1,10)=0.125, p>0.05
(D)	Exploration(%)		F(1,10)=26.555, p<0.05$^{\#}$<br>F(1,10)=5.001, p<0.05*<br>F(1,10)=11.215, p<0.05*
Figure2			
NC vs LPS, LPS vs DP, LPS vs C29, LPS vs DW, LPS vs DC, LPS vs DD			
(B)	Spon. Alternation(%)		F(1,14)=12.868, p<0.05$^{\#}$<br>F(1,14)=4.766, p<0.05*<br>F(1,14)=4.774, p<0.05*<br>F(1,14)=6.633, p<0.05*<br>F(1,14)= 6.142, p<0.05*<br>F(1,14)=10.036, p<0.05*
(C)	Exploration(%)		F(1,14)=23.736, p<0.05$^{\#}$<br>F(1,14)=16.521, p<0.05*<br>F(1,14)=4.752, p<0.05*<br>F(1,14)=4.878, p<0.05*<br>F(1,14)= 14.603, p<0.05*<br>F(1,14)=16.149, p<0.05*
(D)	Latency time(s)	Day1	F(1,14)=0.991, p>0.05<br>F(1,14)=1.051, p>0.05<br>F(1,14)=2.152, p>0.05<br>F(1,14)=2.152, p>0.05<br>F(1,14)= 2.152, p>0.05<br>F(1,14)=0.776, p>0.05
		Day2	F(1,14)=8.932, p<0.05$^{\#}$<br>F(1,14)=4.585, p>0.05<br>F(1,14)=2.273, p>0.05<br>F(1,14)=2.388, p>0.05<br>F(1,14)= 2.946, p>0.05<br>F(1,14)=4.627, p>0.05
		Day3	F(1,14)=12.137, p<0.05$^{\#}$<br>F(1,14)=3.371, p>0.05<br>F(1,14)=0.696, p>0.05<br>F(1,14)=1.091, p>0.05<br>F(1,14)= 5.006, p<0.05*<br>F(1,14)=7.095, p<0.05*
(E)	IL-1β(pg/mg)		F(1,10)=62.962, p<0.05$^{\#}$<br>F(1,10)=9.244, p<0.05*

			F(1,10)=8.018, p<0.05*<br>F(1,10)=10.691, p<0.05*<br>F(1,10)= 11.967, p<0.05*<br>F(1,10)=30.857, p<0.05*
(F)	TNF-α(pg/mg)		F(1,10)=33.332, p<0.05#<br>F(1,10)=5.266, p<0.05*<br>F(1,10)=7.364, p<0.05*<br>F(1,10)=6.639, p<0.05*<br>F(1,10)= 14.220, p<0.05*<br>F(1,10)=42.164, p<0.05*
(G)	IL-10(pg/mg)		F(1,10)=86.939, p<0.05#<br>F(1,10)=45.630, p<0.05*<br>F(1,10)=20.465, p<0.05*<br>F(1,10)=32.982, p<0.05*<br>F(1,10)= 50.505, p<0.05*<br>F(1,10)=45.262, p<0.05*
Figure3			
NC vs LPS, LPS vs DP, LPS vs C29, LPS vs DW, LPS vs DC, LPS vs DD			
(A)	Colon length (cm)		F(1,10)=10.365, p<0.05#<br>F(1,10)=0.058, p>0.05<br>F(1,10)=0.040, p>0.05<br>F(1,10)=1.185, p>0.05<br>F(1,10)= 24.630, p<0.05*<br>F(1,10)=5.929, p<0.05*
(B)	MPO (ng/mg)		F(1,10)=31.666, p<0.05#<br>F(1,10)=16.739, p<0.05*<br>F(1,10)=4.437, p>0.05<br>F(1,10)=7.091, p<0.05*<br>F(1,10)= 32.195, p<0.05*<br>F(1,10)=23.959, p<0.05*
(C)	IL-1β(pg/mg)		F(1,10)=16.770, p<0.05#<br>F(1,10)=5.989, p<0.05*<br>F(1,10)=7.300, p<0.05*<br>F(1,10)=12.019, p<0.05*<br>F(1,10)= 36.937, p<0.05*<br>F(1,10)=12.239, p<0.05*
(D)	TNF-α(pg/mg)		F(1,10)=149.745, p<0.05#<br>F(1,10)=16.416, p<0.05*<br>F(1,10)=6.290, p<0.05*<br>F(1,10)=10.839, p<0.05*<br>F(1,10)= 25.692, p<0.05*<br>F(1,10)=17.187, p<0.05*
(E)	IL-10(pg/mg)		F(1,10)=124.999, p<0.05#<br>F(1,10)=15.751, p<0.05*<br>F(1,10)=8.437, p<0.05*<br>F(1,10)=16.886, p<0.05*<br>F(1,10)= 9.808, p<0.05*<br>F(1,10)=18.415, p<0.05*

Table S2. Effects of orally gavaged donepezil (DON), C29, DW2009 (DW), the mixture of donepezil and C29 (DC) and the mixture of donepezil and DW2009 (DD) on the gut microbiota composition at the phylum level in LPS intraperitoneally injected mice.

Taxon Name	Relative contribution (%)						
	NC	LPS	DON	C29	DW	DC	DD
Actinobacteria	0.06±0.02	0.08±0.03	0.09±0.04	0.07±0.05	0.10±0.05	0.07±0.03	0.12±0.05
Bacteroidetes	67.10±10.65	62.32±9.62	62.99±4.16	57.72±17.09	47.00±5.08*	52.54±10.45	48.90±5.31*
Cyanobacteria	0.20±0.12	1.59±1.76	0.28±0.16	0.46±0.31	0.56±0.86	0.69±0.61	0.59±0.82
Firmicutes	25.94±8.17	28.57±6.92	30.53±5.16	34.15±15.18	44.21±6.81*	40.01±10.64*	44.28±6.99*
Proteobacteria	5.79±3.18	6.94±3.20	4.36±1.77	6.65±3.41	6.95±2.50	6.18±3.34	5.56±2.33
Tenericutes	0.69±0.52	0.36±0.18	1.46±1.60	0.44±0.34	0.24±0.13	0.21±0.17	0.12±0.08*

Values indicate means±SD. **p*<0.05 vs. LPS group.

Table S3. Effects of orally gavaged donepezil (DON), C29, DW2009 (DW), the mixture of donepezil and C29 (DC) and the mixture of donepezil and DW2009 (DD) on the gut microbiota composition at the family level in LPS intraperitoneally injected mice.

Taxon Name	Relative contribution (%)						
	NC	LPS	DON	C29	DW	DC	DD
Bacteroidaceae	7.11±3.77	4.08±1.99	7.59±5.40	4.70±2.35	1.19±0.63*	4.63±2.70	3.30±1.28
Desulfovibrionaceae	0.70±0.44	1.45±0.64#	0.83±0.87	1.47±1.06	1.36±0.55	1.58±1.05	1.16±0.56
Helicobacteraceae	4.24±3.28	5.06±2.76	3.21±0.90	4.34±2.45	5.32±2.12	4.45±2.61	4.22±2.12
Lachnospiraceae	16.44±7.46	18.35±6.51	14.79±5.71	26.58±15.60	30.08±9.34*	31.34±11.05*	30.93±6.76*
Lactobacillaceae	2.66±2.20	2.73±2.25	9.41±8.17	1.63±1.44	6.40±4.53	2.03±1.43	4.77±2.80
Muribaculaceae	39.70±6.59	33.48±11.83	36.06±559	34.31±12.52	33.97±8.49	33.92±8.53	33.75±6.82
Prevotellaceae	13.55±8.00	18.38±8.56	11.95±4.96	13.92±8.72	8.72±7.30*	7.60±4.34*	6.41±3.50*
Rhodospirillaceae	0.70±0.85	0.31±0.22	0.18±0.18	0.66±0.87	0.19±0.13	0.06±0.06*	0.10±0.10*
Rikenellaceae	5.01±1.92	4.14±2.06	4.64±1.36	3.01±0.78	2.37±1.75	3.90±2.41	4.02±1.59
Ruminococcaceae	5.98±2.51	6.28±1.70	5.19±3.17	4.75±2.43	6.93±1.41	5.96±2.24	7.67±1.57

Values indicate means±SD. #*p*<0.05 vs. NC group. **p*<0.05 vs. LPS group.

Table S4. Effects of orally gavaged donepezil (DON), C29, DW2009 (DW), the mixture of donepezil and C29 (DC) and the mixture of donepezil and DW2009 (DD) on the gut microbiota composition at the genus level in LPS intraperitoneally injected mice

Taxon Name	Relative contribution (%)						
	NC	LPS	DON	C29	DW	DC	DD
Alistipes	4.32±1.93	3.45±1.88	4.09±1.37	2.55±0.71	1.85±1.48	3.27±2.43	3.19±1.49
Bacteroides	7.10±3.76	4.07±1.98	7.58±5.39	4.68±2.34	1.19±0.63*	4.63±2.70	3.30±1.28
Helicobacter	4.23±3.28	5.06±2.76	3.21±0.90	4.34±2.45	5.32±2.12	4.45±2.60	4.22±2.12
Lactobacillus	2.65±2.19	2.71±2.23	9.30±8.04	1.63±1.43	6.33±4.47	2.02±1.42	4.74±2.78
Muribaculum	4.40±1.93	3.79±2.24	3.77±1.27	2.50±1.08	6.40±2.39	5.73±2.85	3.65±2.47
Oscillibacter	1.40±1.43	1.27±0.70	0.95±0.52	1.20±0.61	1.97±0.58	1.61±0.58	1.94±0.99
PAC000186_g	10.16±3.40	7.80±3.46	8.42±3.49	8.60±3.60	6.99±2.58	8.25±2.95	8.96±2.28
PAC000198_g	4.11±0.95	3.45±1.41	4.02±1.07	3.07±1.49	2.75±1.40	2.98±0.42	2.97±0.52
PAC000664_g	1.54±1.16	1.76±0.71	2.66±1.82	3.73±2.23*	4.73±2.48*	6.68±4.97*	3.96±1.90*
PAC001068_g	7.67±1.27	5.88±1.91	7.03±2.47	7.02±3.84	7.18±1.38	5.33±2.24	5.81±1.81
PAC001074_g	3.04±1.32	2.31±1.70	3.43±1.14	2.46±0.60	2.15±1.14	2.33±1.94	2.36±1.08
PAC001512_g	1.90±0.74	2.73±1.42	1.54±0.59	1.90±1.11	0.63±0.45*	1.60±1.06	1.87±1.12
Paraprevotella	2.90±4.35	3.70±5.90	3.05±2.79	1.65±1.42	3.73±4.23	1.27±1.58	2.05±1.54
Prevotella	4.07±3.40	6.01±2.93	2.98±1.43*	2.41±1.28*	2.60±1.79*	4.15±2.59	2.25±1.31*
Prevotellaceae_uc	2.68±1.56	7.58±4.75#	5.08±4.66	9.48±8.35	1.97±1.91*	1.24±1.43*	1.43±0.89*

Values indicate means±SD. #*p*<0.05 vs. NC group. **p*<0.05 vs. LPS group.

Table S5. Effects of orally gavaged donepezil (DON), C29, DW2009 (DW), the mixture of donepezil and C29 (DC) and the mixture of donepezil and DW2009 (DD) on the gut microbiota composition at the species level in LPS intraperitoneally injected mice

Taxon Name	Relative contribution (%)						
	NC	LPS	DON	C29	DW	DC	DD
AB599946_s	4.33±2.76	1.90±1.38	3.78±4.05	2.69±1.88	0.62±0.34*	2.39±1.56	1.12±0.53
AB606242_s	0.43±0.46	0.52±0.57	1.11±1.51	1.14±1.40	1.83±2.59	2.25±3.77	1.74±1.13
Bacteroides acidifaciens group	1.42±0.67	1.05±0.66	2.31±1.44	1.11±0.56	0.39±0.19*	1.12±0.87	1.53±0.68
EF097112_s	2.15±0.75	1.21±1.07	2.40±0.70*	2.74±1.49*	2.10±0.87	2.07±1.10	2.23±0.63*
EU622763_s group	2.51±2.05	4.20±1.93	1.33±0.95*	1.36±1.12*	1.64±1.65*	1.87±1.80	1.46±1.09*
FJ880724_s	2.81±4.17	3.67±5.83	3.01±2.75	1.63±1.40	3.69±4.19	1.26±1.57	2.03±1.53
Helicobacter japonicus	1.72±1.28	2.22±1.68	1.12±0.44	2.26±1.25	1.80±1.65	1.05±1.08	2.15±1.56
Helicobacter rodentium group	2.39±3.43	2.65±1.77	1.99±1.09	1.82±1.02	3.33±1.73	3.21±2.40	1.90±0.89
KE159538_g_uc	0.05±0.04	0.03±0.02	1.33±2.30	10.81±16.97	0.18±0.22	6.99±12.60	2.20±5.39
Lactobacillus murinus group	2.28±1.99	1.17±0.84	6.84±6.14*	1.19±1.50	3.05±3.19	1.62±1.23	2.23±2.03
PAC001064_s	3.06±1.22	2.18±1.34	2.25±1.36	2.43±1.82	1.89±1.22	1.63±0.62	3.49±1.06
PAC001065_s group	5.94±2.65	4.17±2.90	4.65±2.40	5.19±2.71	3.03±1.48	4.87±2.36	4.64±1.29
PAC001070_s group	0.75±0.27	1.37±0.87	1.11±1.26	1.40±1.32	1.42±1.29	0.97±0.93	0.44±0.34*
PAC001071_s	1.89±0.66	0.70±0.26$^{\#}$	1.31±1.18	0.86±0.48	1.01±0.68	0.51±0.31	1.44±1.43
PAC001074_s	2.89±1.33	1.72±1.30	2.97±0.89	2.29±0.57	1.36±0.81	0.97±0.82	1.56±0.94
PAC001077_s	2.60±1.83	2.38±1.37	1.15±0.89	0.75±0.55*	3.53±2.06	4.04±2.49	2.05±1.79
PAC001124_s	0.09±0.07	1.51±1.81	0.03±0.04	0.15±0.21	0.58±1.04	0.40±0.40	0.65±0.93
PAC002399_s	2.39±0.67	1.40±0.94$^{\#}$	2.21±0.81	2.29±0.95	2.30±1.03	2.08±1.14	2.74±1.63
PAC002445_s	1.51±0.70	1.60±0.75	1.57±0.63	0.99±0.30	0.41±0.34*	0.81±0.68*	0.53±0.43*
PAC002479_s	3.63±4.46	1.04±1.21	0.82±1.19	0.37±0.61	0.43±0.61	0.92±1.28	0.68±0.59

Values indicate means±SD. $^{\#}p<0.05$ vs. NC group. $^{*}p<0.05$ vs. LPS group.

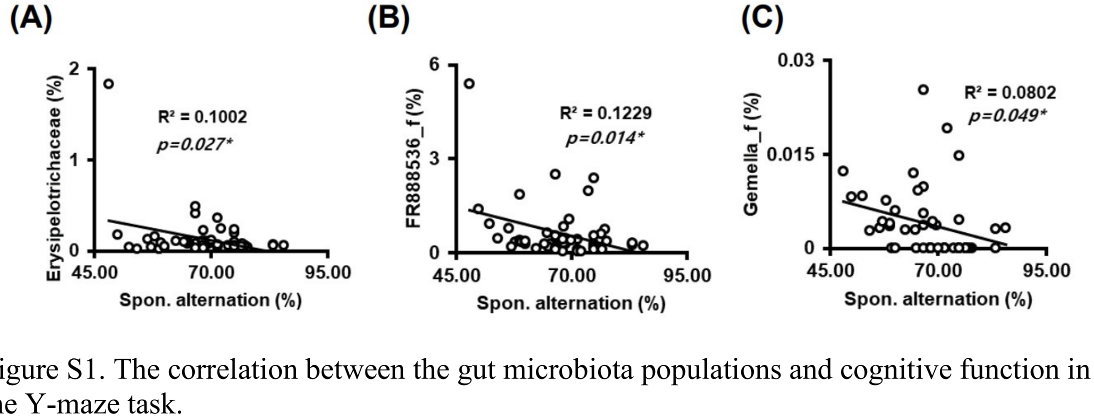

Figure S1. The correlation between the gut microbiota populations and cognitive function in the Y-maze task.
